# Antimicrobial peptides of the Cecropin-family show potent antitumor activity against bladder cancer cells

**DOI:** 10.1186/1471-2490-8-5

**Published:** 2008-03-03

**Authors:** Henrik Suttmann, Margitta Retz, Friedrich Paulsen, Jürgen Harder, Ulrike Zwergel, Jörn Kamradt, Bernd Wullich, Gerhard Unteregger, Michael Stöckle, Jan Lehmann

**Affiliations:** 1 Department of Urology and Pediatric Urology, Saarland University Hospital, Homburg/Saar, Germany; 2 Department of Urology, TU rechts der Isar, München, Germany; 3 Department of Anatomy and Cell Biology, Martin-Luther-Universität Halle-Wittenberg, Germany; 4 Department of Dermatology, University Hospital Schleswig-Holstein, Campus Kiel, Germany

## Abstract

**Background:**

This study evaluated the cytotoxic and antiproliferative efficacy of two well-characterized members of the Cecropin-family of antimicrobial peptides against bladder tumor cells and benign fibroblasts.

**Methods:**

The antiproliferative and cytotoxic potential of the Cecropins A and B was quantified by colorimetric WST-1-, BrdU- and LDH-assays in four bladder cancer cell lines as well as in murine and human fibroblast cell lines. IC_50 _values were assessed by logarithmic extrapolation, representing the concentration at which cell viability was reduced by 50%. Scanning electron microscopy (SEM) was performed to visualize the morphological changes induced by Cecropin A and B in bladder tumor cells and fibroblasts.

**Results:**

Cecropin A and B inhibit bladder cancer cell proliferation and viability in a dose-dependent fashion. The average IC_50 _values of Cecropin A and B against all bladder cancer cell lines ranged between 73.29 μg/ml and 220.05 μg/ml. In contrast, benign fibroblasts were significantly less or not at all susceptible to Cecropin A and B. Both Cecropins induced an increase in LDH release from bladder tumor cells whereas benign fibroblasts were not affected. SEM demonstrated lethal membrane disruption in bladder cancer cells as opposed to fibroblasts.

**Conclusion:**

Cecropin A and B exert selective cytotoxic and antiproliferative efficacy in bladder cancer cells while sparing targets of benign murine or human fibroblast origin. Both peptides may offer novel therapeutic strategies for the treatment of bladder cancer with limited cytotoxic effects on benign cells.

## Background

Depending on grade and stage, non-muscle invasive bladder cancers recur and progress in up to 80% of cases if treated by transurethral resection (TUR) alone[1,2]. Postoperative adjuvant intravesical instillations of chemotherapeutic drugs such as mitomycin, doxorubicin or epirubicin as well as immunotherapy with Bacillus Calmette-Guérin (BCG) are established treatment options to reduce tumor recurrences [3-5]. However, recurrence and progression remain a serious threat to patients especially with high risk non-muscle invasive bladder cancers even if adjuvant intravesical treatment is performed according to existing guidelines with BCG-immunotherapy plus maintenance. In this subgroup of patients, progression occurs in more than 15% of cases [3,5-7]. Furthermore, intravesical chemotherapy and BCG-immunotherapy are both associated with significant side effects. Toxicities vary from mild cystitic symptoms to severe sepsis and have a negative impact on patient compliance [8,9]. Therefore, the identification of new potent intravesical agents is highly desirable to reduce toxicity and to improve long-term outcome.

Cecropin A and B belong to the Cecropin-family of antimicrobial peptides which were first isolated from the hemolymph of the giant silk moth *Hyalophora cecropia *[10-12]. Antimicrobial peptides are important components of the innate immune defense against microbial pathogens in a wide range of organisms including humans [13,14]. Cecropin A and B exert strong antibiotic activity against both Gram-positive and -negative bacteria in micromolar concentrations [10,15]. Cecropins have the ability to form specific amphipathic alpha-helices which allow them to target nonpolar lipid cell membranes. Upon membrane targeting, they form ion-permeable channels subsequently resulting in cell depolarization, irreversible cytolysis and finally death [14,16]. Besides their well-known antimicrobial properties, recent studies have demonstrated specific tumoricidal activity of both Cecropin A and B against mammalian leukemia, lymphoma and colon carcinoma cell lines [17,18] as well as small cell lung cancer [19] and gastric cancer cells [20]. *In vivo*, Cecropin B improves survival of mice bearing ascitic colon adenocarcinomas [18]. Transfection of human bladder cancer cells with Cecropin genes reduces their tumorigenicity in nude mouse models [21]. We have recently reported significant antitumor activity of the structurally and functionally related antimicrobial peptide Magainin II against bladder cancer cell lines *in vitro *[22]. Our aim in this study was to analyze the antitumor efficacy of Cecropins against bladder cancer cells and to evaluate their potential as a new therapeutic option for intravesical treatment of non-muscle invasive bladder cancer.

## Methods

### Peptides, cell lines and cell culture

Lyophilized Cecropin A and B were purchased from Bachem AG (Heidelberg, Germany) and reconstituted in serum-free RPMI 1640 (Sigma) or Dulbecco's modified Eagle medium (DMEM, Gibco BRL), respectively. The four established human bladder cancer cell lines RT4 (grade 1), 647V (grade 2), J82 (grade 3) and 486P (grade 4) were originally obtained from the American Type Culture Collection (ATCC; Rockville, MD). The mouse fibroblast cell line 3T6 was purchased from the German Collection of Microbiology and Cell Culture (DSMZ; Braunschweig, Germany). Primary fibroblasts from human gingival tissue samples were isolated using a standard protocol and termed ZF07 [23]. Cells were cultured under standard conditions as described previously [22]. For cytotoxicity and proliferation assays, cells were seeded in 96-well microtiter plates in a total volume of 200 μl. Cells were cultivated for 24 h before the respective test substances were added after changing the culture medium.

### Cell viability assay

The WST-1 cell viability assay (Roche Molecular Biochemicals; Mannheim, Germany) is based on the cleavage of the tetrazolium salt WST-1 to form a red formazan dye by viable cells and was performed according to the manufacturer's instructions [22]. Cells in microtiter plates were incubated for 24 h with various concentrations of Cecropin A or B as indicated in figure legends. Absorbance of the colored formazan was determined using an automated microplate reader at 450 nm/620 nm wavelength (LambdaE, MWG Biotech, Ebersberg, Germany). The mean absorbance of control wells (cells without Cecropin) represented 100% cell viability. Viability of Cecropin-treated cells was determined in triplicate and related to the absorbance of control cells.

### Cell proliferation assay

Cell proliferation was measured with the BrdU cell proliferation enzyme-linked immunosorbent assay (ELISA) kit (Roche Molecular Biochemicals; Mannheim, Germany) according to the manufacturer's instructions using an automated microplate reader [22]. Cells were incubated for 24 h in various concentrations of Cecropin A or B in microtiter plates at a final volume of 200 μl/well as indicated in figure legends. As a control for the replication of DNA synthesis, cells were cultured without addition of Cecropins. The mean absorbance of control cells represented 100% cell proliferation, and mean absorbance of treated cells was related to control values to determine sensitivity. Cell proliferation (% of control) was determined in triplicate.

### Cell cytotoxicity assay

To detect direct Cecropin-induced cell lysis we performed LDH-release assays (Roche Molecular Diagnostics; Mannheim, Germany) according to the manufacturer's instructions [22]. Target cells were seeded in microtiter plates for 24 h and incubated with various concentrations of Cecropins for another 24 h. To determine the maximum LDH-release, cells were treated with 2% triton-X100 (Sigma; Taufkirchen, Germany) for 10 minutes before running the assay. Untreated cells served as controls for spontaneous LDH-release. Specific LDH-release was calculated according to the following formula: LDH-release % = 100 × (Exp-Spo)/(Max-Spo), with Exp representing experimental release, Spo representing baseline release and Max representing Maximum LDH-release.

### Scanning electron microscopy (SEM)

Clean coverslips were placed at the bottom of two12-well plates. At least 3 × 10^4 ^cells/well were seeded and covered by 1 ml serum-free medium. Plates were incubated overnight and final concentrations of Ceropins were added for 30–90 min the next day as indicated in figure legends. After removing supernatants, 1 ml 4% formalin solution (Sigma; Taufkirchen, Germany) was added to each well. Following fixation, all coverslips were impregnated in 2.5% tannic acid (Sigma; Taufkirchen, Germany) for 2 days. Counter-fixation in 2% osmium tetroxide (Sigma; Taufkirchen, Germany) for 2 hours was followed by dehydration in ethanol and drying in a critical point dryer (Ion Tech Ltd.; Teddington, UK). Cells on coverslips were coated with gold and analyzed using a XL 20 scanning electron microscope (Philips; Kassel, Germany).

### Statistical analysis

All experiments were performed at least in triplicate. The Mann-Whitney U test (Bias 8.1 for Windows) was used to calculate p values. A p < 0.05 was considered statistically significant.

## Results

### Cecropins inhibit bladder tumor cell viability and proliferation

**Figure 1:**
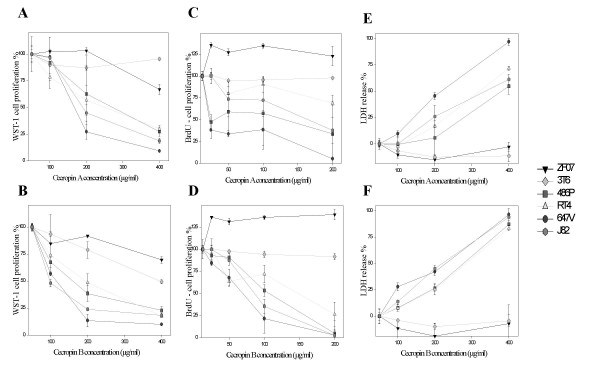
Impact of Cecropin A and B on bladder cancer cells and fibroblasts. The four bladder cancer cell lines RT4, 647V, J82 and 486P as well as human (ZF07) and murine (3T6) fibroblasts were coincubated with increasing concentrations of up to 400 μg/ml Cecropin A and B. Cecropin A (**A**) and B (**B**) both demonstrate significant inhibitory activity on the viability of all four bladder cancer cell lines as measured by the WST-1 cell viability assay. In contrast, benign fibroblasts are less susceptible to Cecropin (p < 0,05). Additionally, Cecropin A (**C**) and B (**D**) both demonstrate significant inhibitory activity on the proliferation of all four bladder cancer cell lines as measured by the BrdU proliferation assay. In contrast, benign fibroblasts are not susceptible to Cecropin (p < 0,05). Cecropin A (**E**) and B (**F**) both exert significant cytotoxicity against all bladder cancer cell lines as measured by the LDH release assay. In contrast, benign fibroblasts are not susceptible to Cecropin-mediated cytolysis (p < 0,05). Experiments were performed in triplicate with results shown as mean ± standard deviation.

The impact of Cecropins A and B on bladder tumor cell viability and proliferation was assessed by WST-1 and BrdU assays. Cecropin A and B both inhibit the viability of bladder cancer cells in a dose-dependent fashion. The average IC_50 _values of Cecropin A and B against all bladder cancer cell lines were 220.05 μg/ml (range 185.39 – 251.47 μg/ml) and 139.91 μg/ml (range 97.93 – 184.81 μg/ml), respectively. In contrast, target cells of benign fibroblast origin such as ZF07 and 3T6 showed significantly higher IC_50 _values in WST-1 assays as compared to bladder tumor targets (p < 0.05). IC_50 _values of Cecropin B against ZF07 and 3T6 cells averaged 573.03 μg/ml (range 413.92 – 732.14 μg/ml) while the IC_50 _of Cecropin A could only be determined against ZF07 cells with 649.03 μg/ml (Fig. 1A + B and Table 1).

**Table 1:** Effect of Cecropin A and B on cell viability, proliferation and cytotoxicity

**Cell line**	**Cell viability** WST-1 IC_50 _(μg/ml) **Cecropin A/B**	**Cell proliferation** BrdU IC_50 _(μg/ml) **Cecropin A/B**	**Cytotoxicity** LDH IC_50 _(μg/ml) **Cecropin A/B**
**ZF07**	649.03/732.14	∅/∅	∅/∅
**3T6**	∅/413.92	∅/∅	∅/∅
**486P**	251.47/161.76	69.2/87.47	373.3/232.4
**RT4**	231.26/184.81	96.22/92.9	289.3/240.4
**647V**	185.39/115.12	28.74/61.86	200.7/181.1
**J82**	212.07/97.93	99.01/77.51	319.2/196.3

IC_50 _values were assessed by logarithmic extrapolation; ∅ = not assessable or concentration >10,000 μg/ml

Cecropin A and B both inhibit BrdU incorporation in all bladder cancer cells tested in a dose-dependent fashion. The average IC_50 _values of Cecropin A and B against all bladder cancer cell lines were 73.29 μg/ml (range 28.74 – 99.01 μg/ml) and 79.94 μg/ml (range 61.86 – 92.9 μg/ml), respectively. Again, in contrast to benign fibroblasts, bladder tumor cells are significantly more susceptible to Cecropin A and B-induced inhibition of proliferation (p < 0.05). IC50 values against ZF07 and 3T6 cells could neither be determined for Cecropin A nor B (Fig. 1C + D and Table 1). In summary, Cecropin A and B both show a selective inhibitory effect on bladder cancer cell viability and proliferation as compared to target cells of benign origin such as fibroblasts.

### Cecropins induce direct tumor cell lysis

Cecropin A and B both induce an increase in LDH-release from bladder cancer cells in a dose-dependent fashion. The direct lytic activity of the Cecropins was slightly less pronounced as compared to their inhibitory effect on tumor cell viability and proliferation. The average IC_50 _of Cecropin A against 486P, RT4, 647V and J82 bladder cancer cell lines was 295.6 μg/ml (range 200.7 – 373.3 μg/ml). The average IC_50 _of Cecropin B against 486P, RT4, 647V and J82 bladder cancer cell lines was 212.6 μg/ml (range 181.1 – 240.4 μg/ml). Bladder tumor cells are significantly more susceptible to Cecropin A and B-induced cytolysis (p < 0.05) than benign fibroblasts. IC50 values against ZF07 and 3T6 cells could neither be determined for Cecropin A nor B (Fig. 1E + F and Table 1). In summary, Cecropin A and B both demonstrate selective cytotoxicity against bladder cancer cells as compared to target cells of benign origin such as fibroblasts.

### Cecropins induce target bladder tumor cell membrane disruption

**Figure 2:**
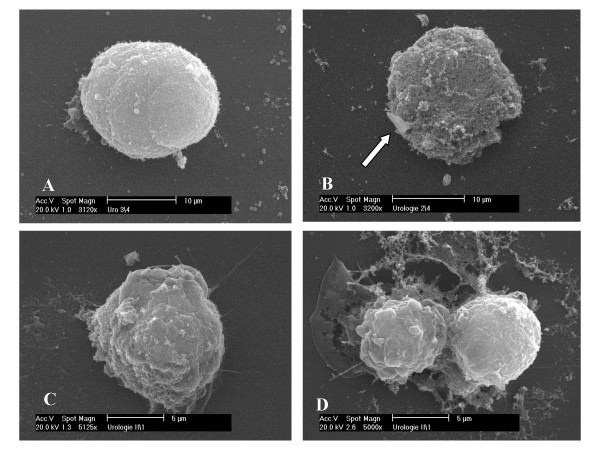
Effect of Cecropin B on cell membranes of 486P bladder cancer cells and ZF07 fibroblasts as visualized by scanning electron microscopy (SEM). **(A) **Representative example of an untreated 486P bladder cancer cell showing a smooth surface. **(B) **486P cells treated with 65 μM Cecropin B reveal a disrupted cell membrane with only small islands of intact surface left (arrow). In contrast, untreated **(C) **ZF07 fibroblasts and (**D**) fibroblasts after incubation with 65 μM Cecropin B do not display any changes in cell morphology with no observable damage to the cell membrane.

Antimicrobial peptides of the Cecropin-family can target nonpolar lipid cell membranes resulting in formation of ion-permeable channels which leads to depolarization, irreversible cytolysis and finally death of the target cells [14,16]. We examined the morphological changes on the cell membrane of 486P bladder tumor cells and ZF07 human gingival fibroblasts induced by Cecropin A and B using scanning electron microscopy (SEM). While untreated 486P cells show a smooth surface, treatment with 65 μM of the most potent peptide Cecropin B induces significant cell membrane disruption. In contrast, untreated and Cecropin B-treated ZF07 cells display unaffected cell morphology without membrane damage (Fig. 2). Cecropin A treatment of tumor cells induces similar, however less pronounced, morphological changes (data not shown).

## Discussion

The innate immune system plays a crucial role in the early defense against malignant tumor growth, and progressing cancers eventually escape immune surveillance [24]. Recently, cytolytic cationic polypeptides which are important components of the innate immune defense against microbial pathogens were demonstrated to overcome this resistance via yet unknown mechanisms [17-20,22,25-27]. The results of the present study demonstrate significant inhibition of tumor cell proliferation and DNA synthesis following treatment with such polypeptides, Cecropin A and Cecropin B, in all bladder cancer cell lines tested. Additionally, both Cecropin A and B exert direct tumor cell lysis, probably by target cell membrane disruption. Interestingly, lytic and antiproliferative action of the Cecropins were restricted to malignant transformed cells whereas benign fibroblast cells were spared from cytotoxicity. Our results support previous findings from studies reporting an antitumor effect of Cecropins. Moore et al. were the first authors who described potent anticancer activity of Cecropin B against a panel of mammalian tumor cells [18]. Various tumor entities such as leukemia, lymphoma, colon carcinoma, small cell lung cancer and gastric cancer have also been described to be sensitive to Cecropin-mediated cell lysis *in vitro *[17-20]. The Cecropins share their potential antitumoral action with structural analogues from related families of antimicrobial peptides such as Magainins or Defensins [22,25]. In the study by Lehmann et al., MagaininII was demonstrated to exert a selective antitumoral potential and even displayed pronounced antiproliferative and cytotoxic activity on high grade as compared to low grade tumor cells [22]. In our experiments, we found a similar trend towards increased susceptibility of high grade tumor cells against cecropins. However, this trend was not statistically significant. The cytolytic mechanism of antimicrobial peptides remains highly controversial. In addition to disrupting the surface membrane of tumor cells which induces cytolysis/necrosis, a second mechanism of action has been implicated for tumor cell destruction: subgroups of antimicrobial peptides are able to enforce disruption of mitochondrial membranes which subsequently leads to activation of apoptosis pathways [25]. However, this finding needs to be confirmed in further studies.

Cecropins and other antimicrobial peptides are especially promising candidates for anticancer therapy in humans because they demonstrate several unique features: 1) Their selectivity for malignant cells and their potentially pronounced lytic activity against high-grade tumor cells allow for an optimal therapy *in vivo *with low therapeutic concentrations and limited side effects. The molecular basis for this selective antitumor activity of antimicrobial peptides has not yet been completely understood. Some authors have suggested that certain physicochemical attributes of the respective target cell membranes such as differences in lipoprotein content or fluidity may explain this phenomenon [28]. Furthermore, serum composition has been shown to have an important impact on the activity of lytic peptides, with high concentrations of albumin and low-density lipoprotein being particularly inhibitory [29] 2) The antitumoral effect of Cecropins has been hypothesized to be unaffected by the multidrug resistance (MDR) phenotype observed in many cancer types. In clinical practice, the continual emergence of drug resistance hinders the activity of most standard anticancer agents. Cecropins as well as other antimicrobial peptides have been demonstrated to have significant lytic activity against MDR cancer cells [18,30] 3) Classic chemotherapeutic agents such as mitomycin which are widely used for intravesical instillation are highly unstable in urine [31]. In contrast, Cecropins are largely resistent against serum and urine proteolysis because of their specific biochemical structures [14], rendering them ideal candidates for intravesical tumor therapy.

Most studies investigating the antitumoral action of Cecropins and other antimicrobial peptides have been performed *in vitro*, but data confirming this promising potential *in vivo *are rare. Presumably, this is due to the fact that highly potent peptides such as Cecropins immediately exert their lytic activity locally at the respective application sites, making them unsuitable for systemic therapy. Consequently, application of Cecropins *in vivo *has been restricted to either hollow organs or anatomically restricted sites such as the abdominal cavity or skin [18,21]. Recently, however, Papo et al. were able to demonstrate that specifically designed related peptides are capable of overcoming many of the limitations associated with their systemic application such as serum proteolysis [27] Additionally, the same group identified a peptide with selective *in vivo *antitumoral activity suitable for systemic application [26]. Still, the urinary bladder represents an ideal target organ where antimicrobial peptides could be administered intravesically, and the Cecropins could act locally on bladder cancer cells.

## Conclusion

In summary, Cecropin A and B exert significant selective cytotoxic and antiproliferative efficacy in bladder cancer cells while sparing targets of benign murine or human fibroblast origin. Their unique mechanism of action appears to depend at least partially on the disruption of target cell membranes resulting in irreversible cytolysis and cell destruction. Both, Cecropin A and B are promising candidates for further preclinical evaluation as intravesical treatment options in non-muscle invasive bladder cancer.

## Competing interests

The author(s) declare that they have no competing interests.

## Authors' contributions

All authors have read and approved the final manuscript.

HS, MR, JL and BW are responsible for the design of the study and the individual experiments, fundraising and the writing of the final manuscript.

FP and JH provided crucial help for initial handling of the peptides and performed the SEM studies.

JK and MS are responsible for statistical analysis, interpretation and presentation of the data.

UZ and GU performed additional cytotoxicity/LDH-release experiments.

## Pre-publication history

The pre-publication history for this paper can be accessed here:


